# Replantation in Pyorrhea Alveolaris

**Published:** 1897-12

**Authors:** Robert Eugene Payne

**Affiliations:** New York


### THE DENTAL REGISTER.

VOL. LI.] DECEMBER, 1897. [No. 12.

### COMMUNICATIONS.

### Replantation in Pyorrhea Alveolaris.

BY ROBERT EUGENE PAYNE, M.D., D.D.S., NEW YORK.

Abstract of a paper read at the Southern Dental Association, Old Point Comfort, Va., August, 1897.

In the history of implantation, transplantation and replantation, the latter has probably been the most successful, possibly because a portion of the membrane remains intact, acting as a scaffolding, or not unlike a sponge graft, in which loops of tissue quickly form, aiding in the formation of a provisional callus, which later, becomes disease bone.

In pyorrhea alveolaris, when the teeth have become very loose and partly pushed out of their sockets, assuming distortions and mal-positions by elongation and mal-occlusion, extreme measures are required to correct the condition.

Believing that I go to the surgical limit in my efforts to effect a cure, I will give in minute detail a description of a case that covers my plan of treatment most thoroughly.

Patient, lady, forty, well-to-do, willing to pay for treatment. Has been under treatment for several years for pyorrhea; informed that three upper front teeth must be lost. Refused to wear plate; teeth wired in; exhibits casts showing marked mal-occlusion; upper and lower front teeth crowded between canines; long lower lateral laps over the canine and pushes the upper central and lateral out, making a deformity so prominent as to have been made a feature in passport discription. The upper right central and lateral and left bicuspid are elongated and sockets almost obliterated. Five dead pulps, gums congested, painful and discharging pus. Two dead pulps in the molars were removed, canals sterilized with 25 percent pyrozone, placing the thumb over the opening, driving it through the root and sterilizing beyond the apex. Roots filled with parafine and salol.

The next step was removal of calculus from roots, taking three teeth at a sitting, using Rigg's and Younger's scalers, applying in the bottom of the sockets a 2 percent cocain solution containing one-third glycerin and a few drops iodin, obtaining a very perfect anesthesia. Pockets washed out with warm boiled water and a drop or two of 25 percent pyrozone introduced. Wash this out, dry the gum and pocket, as far as possible, and with minim syringe place two or three drops concentrated lactic acid at the bottom of the pockets. Then apply each time 3 percent pyrozone as prescribed for use on the tooth-brush night and morning and as mouth-wash during the day.

This was repeated on other teeth successively until all the calculus was thoroughly removed from all the teeth and all the pockets treated. The next thing to receive attention is the crowded lower incisors which are made narrower, the cutting edges evened up and all drawn into position by means of a C. & D. silk twist, removed two or three times a week. If tied carefully and taut this will shrink, drawing the teeth in place. This was effected in two weeks when they were splinted by being bound together with "00" silk twist, waxed and secured with square knots. The next step was to remove the three upper loose teeth, which could have been done with the fingers. The pulps were dead in all. The teeth were sterilized, contents of pulp chambers removed, root canals filled with parafine and salol. The gum on either side of the sockets was then injected with five minims 2 percent cocain solution, the sockets sterilized with pyrozone, and with Ottolengui reamer the socket deepened sufficiently to receive the teeth. The bony tissue in the bottom of the partially obliterated sockets was very dense. The four upper incisors were also reduced in size to get these in the arch between the canines. To make sure that replanted teeth are surgically clean, a few fine crystals of Resorcin are placed on the apex of the root just before placing it in the socket. These crystals dissolve readily and the wound heals quickly with very slight soreness. These teeth were splinted for thirty days and then a gold plate applied, swaged to fit over the cutting edges, as shown in model. The essential points in the treatment are removal of dead pulps and filling roots, correcting irregularities and malocclusion (in this case by reducing the size of teeth), deepening sockets and replanting elongated loosened teeth, removal of calculus, treatment of pockets, splinting. Above all, surgical cleanliness throughout. Special attention was called to the use of Resorcin crystals at the apex of root of implanted teeth. The teeth became firm in from thirty to sixty days after splinting

DISCUSSION.

Dr. Gordon White said that with patience and perseverance on the part of the dentist, and with the co-operation of the patient, the usefulness of teeth even very seriously affected with pyorrhea, could be prolonged many years, but he had never known any cases of positive cure; he does not believe that it can ever be entirely eradicated. He has the best results from the lactic-acid treatment introduced by Dr. Younger, though when the roots of the teeth are very sensitive, he uses the lactate of silver. It does not dissolve in less than fifteen times its own weight in water, so that if the dry powder is placed in the pocket, it acts as a foreign body and increases the irritation. If the deposits are thoroughly removed the secretion of pus will be stopped and the gum will contract so closely that it is impossible to pass the finest instrument between the gum tissue and the side of the tooth without causing severe pain. It may not be technically a *union*, but practically it is so.

Dr. John S. Marshall criticised the use of the terms secretion and excretion, as applied to the formation of pus, which is a product, the result of the death of leucocytes. When asked for a more correct term, Dr. Marshall replied—the word formation covers it.

Dr. H. E. Beach: There is a vast difference between checking the progress of a disease and eradicating it. I do not think the treatment has yet been found that will make a radical cure of pyorrhea though I hope to see the day when it will be done.

Dr. J. P. Corley: There is one sure cure for pyorrhea and that is to extract the tooth. What does Dr. Beach consider a cure? Is it to absolutely obliterate the footprints of the disease?

Dr. Beach: It must be eradicated to the extent that it does not return within two years. Ten years ago I offered a reward of a thousand dollars for a case coming within that limit, but the reward has not yet been claimed.

Dr. Corley: If the physician was required to keep his patients well for two years after curing them of typhoid fever I do not believe we would have many doctors.

Dr. J. S. Marshall spoke at some length of senile changes as a factor in the etiology of pyorrhea alveolaris, the teeth dropping out as the hair falls out, both teeth and hair being dermal appendages. As the falling out of the hair is apparently due to a senile change in the bulbs of the hair, so also the loosening of the teeth in pyorrhea may be the result of senile changes affecting the membranes around the roots of the teeth. He said: "I do not claim this as *the cause*, but I believe it is one of the causative factors in this disease.

Dr. C. N. Peirce elucidated at some length the uric-acid diathesis theory, in true pyorrhea, the system being loaded with waste products, causing a congestion at the apical spaces with an exudation of lime salts deposited at the apical end of the root, from the serum, before there is any distinctive action at the gingival border.

